# Medicines for the Treatment of Obesity

**DOI:** 10.3390/ijms22083866

**Published:** 2021-04-08

**Authors:** Soonshik Shin, Michung Yoon

**Affiliations:** 1 Department of Formula Sciences, College of Korean Medicine, Dongeui University, Busan 47227, Korea; ssshin@deu.ac.kr; 2 Department of Biomedical Engineering, Mokwon University, Daejeon 35349, Korea

Obesity is the result of an energy imbalance caused by an increased ratio of caloric intake to energy expenditure. In conjunction with obesity, related metabolic disorders, such as type 2 diabetes mellitus, dyslipidemia and hypertension, have become global health problems [1]. Reducing body weight by lifestyle modification is recommended, but drug intervention is necessary for morbidly obese individuals. Four drug therapies, including orlistat, naltrexone/bupropion, phentermine/topiramate and liraglutide, were approved for long-term use by the U.S. Food and Drug Administration for the treatment of obesity [2,3,4]. Several other dietary supplements for weight loss are currently sold on a large scale. These supplements contain a variety of ingredients, such as herbs and their active components, dietary fiber, caffeine and minerals [5,6,7]. So far, many antiobesity drugs have been developed but have not been approved for long-term use due to serious adverse side effects or a lack of sufficient efficacy, leading to the need for new antiobesity drugs that are effective and have no side effects. In this Special Issue [8], we have published experimental papers and a review article showing significant findings in the field of medicines for the treatment of obesity.

In studies using cell and animal models, Jeong and Park [9] provided interesting evidence that ergosterol peroxide from the medicinal mushroom *Ganoderma lucidum* inhibits differentiation and lipid accumulation of 3T3-L1 adipocytes via reducing the expression of genes responsible for lipogenesis and the phosphorylation of mitogen-activated protein involved in cell proliferation and differentiation and is, thus, a promising natural agent for obesity and related metabolic diseases. Lee et al. [10] showed that gomisin N from *Schisandra chinensis* ameliorates lipid accumulation and induces a brown fat-like phenotype through AMP-activated protein kinase in 3T3-L1 adipocytes. The authors also described that gomisin N inhibits adipogenesis and lipogenesis by enhancing fatty acid oxidation and thermogenesis and may have a potential preventive and therapeutic agent to combat obesity. Lee et al. [11] demonstrated that lemon balm extract regulates obesity and improve insulin sensitivity via activation of hepatic peroxisome proliferator-activated receptor α (PPARα) in high-fat diet-fed obese C57BL/6J mice. The authors described that lemon balm extract reduces lipid accumulation and stimulates PPARα reporter gene expression in HepG2 cells.

In a human study, Auguet et al. [12] reported that high circulating levels of interleukin-8 (IL-8) are associated with the diagnosis of nonalcoholic steatohepatitis (NASH) in women with morbid obesity. Moreover, there were positive relationships between circulating levels of interleukin-8 and hepatic expression of toll-like receptors. The authors suggest that circulating IL-8 may be a noninvasive biomarker of NASH in morbidly obese women.

In a recent review, Kuryłowicz and Puzianowska-Kuźnicka [13] summarized the current knowledge of adipose tissue browning as an antiobesity strategy. The authors described the types of adipose tissue and their function, adipose tissue browning and its mechanism and pharmacological and non-pharmacological interventions, aiming at white adipose tissue browning and brown adipose activation. The browning strategies of white adipose tissues against obesity are effective in obesity models of cells and animals, but few of them seem to be applicable in humans. Therefore, the authors suggest that brown adipose tissue activation and potential effects should be further studied in order for adipose tissue browning to be applicable therapeutic targets for humans.

This Special Issue offers interesting findings from cell, animal and human studies on medicines for the treatment of obesity and suggests strategies for the development of future antiobesity therapies.

## Data Availability

Not applicable.

## References

[1] Després J.P., Lemieux I. (2006). Abdominal obesity and metabolic syndrome. Nature.

[2] Bray G.A., Ryan D.H. (2012). Medical therapy for the patient with obesity. Circulation.

[3] Valentino M.A., Lin J.E., Waldman S.A. (2010). Central and peripheral molecular targets for antiobesity pharmacotherapy. Clin. Pharmacol. Ther..

[4] Krentz A.J., Fujioka K., Hompesch M. (2016). Evolution of pharmacological obesity treatments: Focus on adverse side-effect profiles. Diabetes Obes. Metab..

[5] Pittler M.H., Ernst E. (2004). Dietary supplements for body-weight reduction: A systematic review. Am. J. Clin. Nutr..

[6] Saper R.B., Eisenberg D.M., Phillips R.S. (2004). Common dietary supplements for weight loss. Am. Fam. Physician..

[7] Watanabe M., Risi R., Masi D., Caputi A., Balena A., Rossini G., Tuccinardi D., Mariani S., Basciani S., Manfrini S. (2020). Current evidence to propose different food supplements for weight loss: A comprehensive review. Nutrients.

[8] Medicines for the Treatment of Obesity. Special Issue. https://www.mdpi.com/journal/ijms/special_issues/Medicines_Obesity#published.

[9] Jeong Y.U., Park Y.J. (2020). Ergosterol peroxide from the medicinal mushroom ganoderma lucidum inhibits differentiation and lipid accumulation of 3T3-L1 adipocytes. Int. J. Mol. Sci..

[10] Lee K., Lee Y.J., Kim K.J., Chei S., Jin H., Oh H.J., Lee B.Y. (2020). Gomisin N from schisandra chinensis ameliorates lipid accumulation and induces a brown fat-like phenotype through amp-activated protein kinase in 3T3-L1 adipocytes. Int. J. Mol. Sci..

[11] Lee D., Shin Y., Roh J.S., Ahn J., Jeoong S., Shin S.S., Yoon M. (2020). Lemon balm extract ALS-L1023 regulates obesity and improves insulin sensitivity via activation of hepatic PPARα in high-fat diet-fed obese C57BL/6J mice. Int. J. Mol. Sci..

[12] Auguet T., Bertran L., Binetti J., Aguilar C., Martínez S., Sabench F., Lopez-Dupla J.M., Porras J.A., Riesco D., Del Castillo D. (2020). Relationship between IL-8 Circulating Levels and TLR2 Hepatic Expression in Women with Morbid Obesity and Nonalcoholic Steatohepatitis. Int. J. Mol. Sci..

[13] Kuryłowicz A., Puzianowska-Kuźnicka M. (2020). Induction of adipose tissue browning as a strategy to combat obesity. Int. J. Mol. Sci..

